# Establishing the prevalence of common tissue‐specific autoantibodies following severe acute respiratory syndrome coronavirus 2 infection

**DOI:** 10.1111/cei.13623

**Published:** 2021-06-13

**Authors:** Alex G. Richter, Adrian M. Shields, Abid Karim, David Birch, Sian E. Faustini, Lora Steadman, Kerensa Ward, Timothy Plant, Gary Reynolds, Tonny Veenith, Adam F. Cunningham, Mark T. Drayson, David C. Wraith

**Affiliations:** ^1^ Clinical Immunology Service Institute for Immunology and Immunotherapy University of Birmingham Birmingham UK; ^2^ Institute of Immunology and Immunotherapy University of Birmingham Birmingham UK; ^3^ Department of Critical Care Medicine University Hospitals Birmingham NHS Trust Birmingham UK

**Keywords:** autoantibodies, autoimmunity, COVID‐19, long COVID, SARS‐CoV‐2

## Abstract

Coronavirus 19 (COVID‐19) has been associated with both transient and persistent systemic symptoms that do not appear to be a direct consequence of viral infection. The generation of autoantibodies has been proposed as a mechanism to explain these symptoms. To understand the prevalence of autoantibodies associated with severe acute respiratory syndrome coronavirus 2 (SARS‐CoV‐2) infection, we investigated the frequency and specificity of clinically relevant autoantibodies in 84 individuals previously infected with SARS‐CoV‐2, suffering from COVID‐19 of varying severity in both the acute and convalescent setting. These were compared with results from 32 individuals who were on the intensive therapy unit (ITU) for non‐COVID reasons. We demonstrate a higher frequency of autoantibodies in the COVID‐19 ITU group compared with non‐COVID‐19 ITU disease control patients and that autoantibodies were also found in the serum 3–5 months post‐COVID‐19 infection. Non‐COVID patients displayed a diverse pattern of autoantibodies; in contrast, the COVID‐19 groups had a more restricted panel of autoantibodies including skin, skeletal muscle and cardiac antibodies. Our results demonstrate that respiratory viral infection with SARS‐CoV‐2 is associated with the detection of a limited profile of tissue‐specific autoantibodies, detectable using routine clinical immunology assays. Further studies are required to determine whether these autoantibodies are specific to SARS‐CoV‐2 or a phenomenon arising from severe viral infections and to determine the clinical significance of these autoantibodies.

## Graphical abstract

Acute infection with COVID is associated with a pattern of autoantibodies including a high proportion with epidermal antibodies. This autoantibody pattern is more common in severe COVID and is persistent up to 6 months.

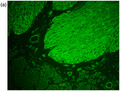

## INTRODUCTION

Infection is a common event that can disrupt immunological tolerance and, in some circumstances, lead to autoimmune disease (1). Viral infections have been linked to both the initiation of a range of autoimmune diseases and disease relapse in individuals with existing conditions (2). For most autoimmune diseases, it is not clear whether infection is the sole precipitating event, an inevitable consequence of a genetic predisposition or whether infection is a necessary trigger in a genetically susceptible individual.

Early data suggest that autoimmune phenomena may exacerbate the immune pathology associated with severe acute respiratory syndrome coronavirus 2 (SARS‐CoV‐2) infection or trigger long‐term autoimmune complications secondary to bystander activation or molecular mimicry. There are reports of SARS‐CoV‐2 infection being associated with a number of autoimmune disorders, including Guillain–Barre syndrome (GBS) (3) and various cytopenias (4). Anti‐phospholipid antibodies have been detected in ~50% of hospitalized patients and linked to an increased incidence of cerebral infarction; however, the clinical relevance of this observation in COVID‐19 remains controversial, as anti‐phospholipid antibody generation in acute illness is a common, non‐specific finding (5, 6, 7). Also, neutralizing antibodies against type 1 anti‐viral cytokines, interferon (IFN)‐ω and/or IFN‐α have been found in more than 10% of patients with coronavirus 19 (COVID‐19) pneumonia (8). By screening a yeast expression library, Wang *et al*. identified autoantibodies against cytokines (including type 1 IFNs), central nervous system (CNS) antigens and extracellular matrix proteins whose frequency correlated with disease severity (9).

Paediatric multi‐system inflammatory syndrome (PIMS‐TS) is a rare condition that occurs as a late complication of SARS‐CoV‐2 infection. Children suffering from this post‐COVID 19 inflammatory condition were also seropositive for anti‐endothelial antibodies that may contribute to their pathology (10, 11, 12). We and others are now searching for tissue specific autoantibodies in adults. Kreye and colleagues screened for CNS autoantibodies using murine brain sections identifying anti‐Yo and N‐methyl‐D‐aspartate receptor (NMDA‐R) as well as a variety of antibodies against epitopes, including vessel endothelium (12). Evidence has emerged that some of these antibodies may arise through cross‐reactive recognition of self‐antigens by antibodies specific for SARS‐CoV‐2 (13).

Identifying the relationship between autoimmune antibody induction and COVID‐19 is further complicated by the spectrum of presentations of this disease. In particular, the severity of disease may require hospitalization and the more severe presentations of disease may influence the maintenance of autoantibodies during convalescence. To investigate any potential links between SARS‐CoV‐2 infection and autoantibodies we examined sera from acute and convalescent COVID‐19 patients, some of whom had been hospitalized, for the presence of autoantibodies to a spectrum of antigens by indirect immunofluorescence. We identify a high frequency and wide range of clinically relevant autoantibodies in both acute and convalescent samples from COVID‐19 patients. Their frequency and tissue specificity suggest that autoantibodies may contribute to the long‐term consequences of COVID‐19.

## METHODS

### Participants

Four cohorts of participants were recruited (Table 1). The first group included 32 individuals sampled during their stay on the ITU for reasons other than COVID‐19 to determine whether acute critical illness per se was associated with autoantibody production; 56% of this cohort were admitted to ITU secondary to infective pathology, most commonly pneumonia. The second group included 25 individuals who were sampled during their stay on the intensive therapy unit (ITU) following a diagnosis of severe COVID‐19. The third group included 35 individuals who had been admitted to ITU with COVID‐19, survived and were sampled 3‐6 months later during routine outpatient follow up. This group explored persistence of any potential antibodies. The fourth group included 24 convalescent health care workers sampled one to three months after mild to moderate COVID‐19 that did not require hospitalisation to determine whether disease severity makes a difference to the generation of any autoantibodies.

### Autoantibody assays

A broad spectrum of anti‐neutrophil and organ‐specific autoantibodies were investigated in serum samples by indirect immunofluorescence. The assays undertaken included the full range of autoimmune tests available in an accredited ISO 15189:2012 National Health Service (NHS) Clinical Immunology laboratory. The full list of assays, manufacturers and disease association are described in Supporting information, Table S2; in short, we undertook indirect immunofluorescence using commercial pre‐prepared slides to detect immunoglobulin (Ig)G antibodies for adrenal, autoimmune encephalitis, anti‐neutrophil (ANA), anti‐neutrophil cytoplasmic antibodies (ANCA), cardiac, epidermal, islet cell, a range of cerebellar (Purkinje cell) antibodies, smooth muscle, mitochondrial, gastric parietal cell, skeletal muscle and endomysial antibodies. The Ig isotype detected was IgG, with the exception of endomysial antibodies, which are IgA. All samples were read by two experienced clinical laboratory scientists for agreement. Results are presented as a qualitative assessment describing the presence or absence of specific autoantibodies in each sample; additional staining findings were descriptive. Intergroup comparison was made by χ^2^ test using GraphPad Prism version 9.

## RESULTS

There were 116 patient samples tested; 32 from group 1 (acute, non‐COVID‐19, ITU), 25 from group 2 (acute, COVID‐19, ITU), 35 from group 3 (convalescent, COVID‐19, post‐ITU) and 24 from group 4 (convalescent, COVID‐19, non‐hospitalized). The demographic descriptions in Table 1 found a female preponderance and slightly younger cohort in the non‐hospitalized convalescent group. The average time from symptom onset was longer in the post‐ITU patients (151 days) than the non‐hospitalized convalescent cohort (38 days). Non‐white ethnicity is higher in all the COVID groups than the non‐COVID ITU cohort, in keeping with known risk factors for severe COVID (14). The details of number of samples tested are described in Table 2; for some patients there was insufficient sample material to run all tests.

**TABLE 1:** Demographics of study groups

Group	Disease	*N*	Age (years)	Female, *n* (%)	Non‐white ethnicity, *n* (%)	Days from symptom onset	Prior known autoimmune disease *n* (%)	Treated with corticosteroids *n* (%)	Survived, *n* (%)
1	Non‐COVID‐19 (severe, acute, ITU admission)	32	62.0 (49.0–74.0)	15 (47)	7 (22)	NA	4 (13)	5^*^ (15)	22 (69)
2	COVID‐19 (severe, acute, ITU admission)	25	55.0 (49.50–62.00)	4 (16)	12 (48)	20 (14.0–23.5)	3 (12)	1 (4)	17 (68)
3	COVID‐19 (prior ITU admission, recovered)	35	54.0 (47.0–62.0)	6 (17)	17 (49)	151 (117–204)	3 (9)	5 (14)	35 (100)
4	COVID‐19 (ambulatory non‐hospitalized, recovered)	24	45.0 (35.25–50.75)	18 (75)	8 (33)	38 (32.0–44.5)	0 (0)	0 (0)	24 (100)

Note

Median and interquartile ranges are provided for continuous variables. Participants in group 4 correspond to individuals with scores of 1 or 2 on the World Health Organization (WHO) coronavirus 19 (COVID‐19) ordinal severity scale. Participants in group 2 correspond to individuals with scores of > 5 on the WHO COVID‐19 ordinal severity scale. ^*^One additional patient was receiving low‐dose hydrocortisone replacement therapy.

Abbreviations: ITU, intensive care unit; NA, not applicable.

**TABLE 2:** Prevalence of tissue‐specific autoantibodies in study groups

Tissue	Isotype	Group 1 (acute, non‐COVID‐19, ITU)			Group 2 (acute COVID‐19, ITU)			Group 3 (convalescent, COVID‐19, post‐ITU)			Group 4 (convalescent, COVID‐19, non‐hospitalized)		
		Positive	Total	**%**	Positive	Total	**%**	Positive	Total	**%**	Positive	Total	**%**
Adrenal	IgG	1	28	3.6	0	22	0.0	0	36	0.0	0	24	0.0
Autoimmune encephalitis screen	IgG	2	21	9.5	1	25	4.0	0	36	0.0	0	24	0.0
ANA	IgG	3	30	10.0	4	25	16.0	1	36	2.8	0	24	0.0
ANCA	IgG	1^‡^	30	3.3	2	25	8.0	3	36	8.3	2	24	8.3
Cardiac	IgG	2	21	9.5	1	14	7.1	10	36	27.8***	0	24	0.0
Endomysial	IgA	0^§^	30	0.0	0^¶^	22	0.0	0	36	0.0	0	24	0.0
Epidermal (IC)	IgG	3	30	10.0	9	22	40.9*	7	36	19.4	6	24	25.0
Epidermal (BM)	IgG	0	30	0.0	0	22	0.0	3	36	8.3	0	24	0.0
Islet cell antibodies	IgG	1	26	3.8	0	25	0.0	1	36	2.8	0	24	0.0
Purkinje cell antibodies	IgG	0	25	0.0	1^‡‡^	22	4.5	0^††^	36	0.0	0	24	0.0
Smooth muscle antibodies	IgG	2	27	7.4	0	25	0.0	11	36	30.6^†^	4	24	16.7
Gastric parietal cell antibodies	IgG	2	27	7.4	0	25	0.0	0	36	0.0	1	24	4.2
Skeletal muscle antibodies	IgG	1	25	4.0	4	24	16.7	7	36	19.4**	0	24	0.0

Abbreviations: BM, basement membrane; IC, intracellular; ITU, intensive therapy unit.

* *p* = 0.01 (group 2 *versus* group 4)

** *p* = 0.02 (group 2 *versus* group 3)

*** *p* = 0.005 (group 3 *versus* group 4).

† *p* = 0.006 (χ^2^ comparing all groups).

‡ Patient with known granulomatosis with polyangiitis (cANCA‐positive).

§ Two patients demonstrated positive immunoglobulin (Ig)A intercellular antibody pattern despite being negative for endomysial antibodies.

¶ Eight patients demonstrated positive IgA intercellular antibody pattern despite being negative for endomysial antibodies.

†† One patient had intense molecular layer staining and one patient had white matter layer staining – both patterns do not have a known disease association.

‡‡ Patient had a positive SOX‐1 staining pattern.

**FIGURE 1:**
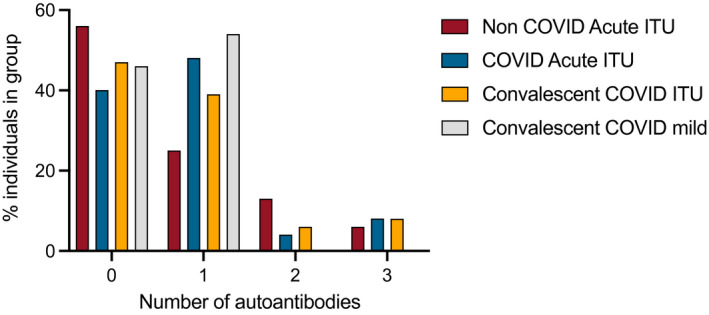
Frequency and quantity of autoantibodies in each cohort

The number of autoantibodies varied between the groups. The highest numbers of autoantibodies to different antigenic targets was detected in the severe COVID disease groups. In group 1 (acute non‐COVID‐19, ITU), 13 of 32 (41%) individuals had autoantibodies; eight tested positive for one autoantibody, four for two autoantibodies and one for three. For group 2 (acute COVID‐19, ITU), 15 of 25 (60%) individuals had autoantibodies; 12 tested positive for one autoantibody, one for two autoantibodies and two for three. In group 3 (convalescent COVID‐19, post‐ITU), 27/36 (77%) individuals had autoantibodies, 14 tested positive for one autoantibody, 10 for two autoantibodies and three for three. In group 4 (convalescent, COVID‐19, non‐hospitalized), 13 of 24 (54%) individuals had autoantibodies and none tested positive for more than one (Figure 1).

**FIGURE 2:**
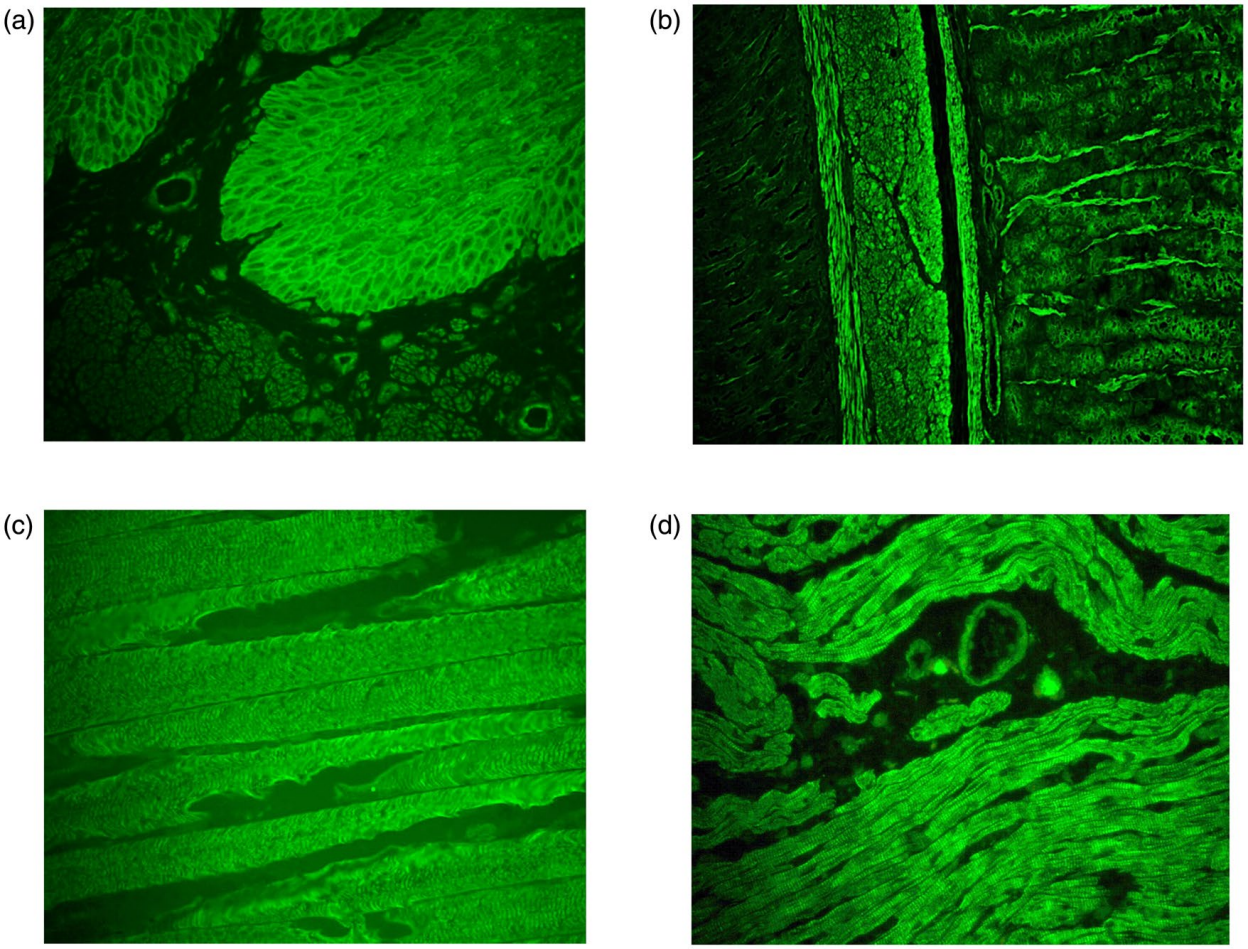
Tissue‐specific staining patterns following coronavirus 19 (COVID‐19). (a) Intracellular cement staining pattern, weak positive (P24), (b) smooth muscle staining pattern, 1/100 titre (P85), (c) skeletal muscle staining pattern, weak positive (P106), (d) cardiac muscle staining pattern demonstrating striations (P87)

Representative slides of epidermal, skeletal, cardiac and smooth muscle autoantibodies are shown in Figure 2. In the acute non‐COVID‐19 ITU patients there were many different causes of their illness (Supporting information, Table S1) and autoantibodies were found against nearly all (12 of 13) the different autoantigens examined, indicating a more random distribution. A higher proportion of acute COVID‐19 ITU patients had autoantibodies (60 *versus* 41%), but to a narrower range of autoantigens (seven of 13) with a preponderance of epidermal (41%) and skeletal antibodies (17%). This preponderance was seen in convalescent, COVID‐19, post‐ITU with epidermal (19%) and skeletal antibodies (19%), but additionally cardiac muscle antibodies (28%) and smooth muscle antibodies (31%). Representative slides of epidermal, smooth muscle, skeletal muscle and cardiac muscle autoantibodies are shown in Figure 2.

In the convalescent, COVID‐19, non‐hospitalized cohort, fewer individuals had autoantibodies than the convalescent ITU cohort (54 *versus* 75%) and against only four autoantigens: epidermal (25%), smooth muscle (17%), ANCA (8%) and gastric parietal (4%). These results suggest that COVID‐19 infection is associated with autoantibody induction, and that these antibodies target a limited repertoire of self‐antigens.

## DISCUSSION

SARS‐CoV‐2 is associated with a spectrum of illness during the acute viral infection, persisting during convalescence and as part of the long COVID syndrome. Constitutional, respiratory, cardiac, neurological, musculoskeletal and psychiatric symptoms are being increasingly described, but the mechanisms behind these are uncertain (15). It is not known whether these phenomena arise as a direct effect of the virus or from off‐target immune effects, including autoimmunity. Our study found that there is a high prevalence of autoantibodies found in the acute and convalescent phase of COVID‐19, suggesting that SARS‐CoV‐2 infection is associated with significant perturbations of immunological tolerance and raising the possibility that autoimmunity may play a role in the pathogenesis of acute and chronic symptoms.

Two‐fifths of the acute non‐COVID‐19 ITU patients had autoantibodies, suggesting that acute severe illness *per se* is associated with autoantibody production, and the wide range of target autoantigens may reflect the diversity of this cohorts’ underlying disease. Three‐fifths of the acute COVID‐19 ITU patients had autoantibodies and these were of a narrower diversity, with antibodies against epidermal intercellular cement and skeletal muscle predominant. These antibodies were persistent over time and also detected in convalescence post‐ITU COVID‐19, more than 5 months from symptoms onset. In addition, cardiac and smooth muscle antibodies were identified. Cardiac and skeletal muscle autoantibodies were not found in convalescent individuals with non‐hospitalized COVID‐19, although smooth muscle antibodies were detected, and a quarter had antibodies directed at epidermal intercellular cement.

The link between infection and autoimmunity is well described, with multiple genetic and environmental factors implicated (1). Pathogenic mechanisms elucidated include molecular mimicry, epitope spreading, revelation of cryptic antigen and bystander activation, although which specific mechanism occur in which situation is usually uncertain. Similarly, just because an autoantibody is generated does not necessarily mean that the autoantibody is pathogenic. In some conditions such as myasthenia gravis there is a clear link between acetyl choline receptor antibodies and dysfunction of the motor end plate, whereas in some conditions such as systemic lupus erythematosus the presence of high‐titre ANA is a non‐specific biomarker of disease and the autoantibodies are not thought to be pathogenic. One of the limitations in understanding the role of autoantibodies in infectious disease has been the relative paucity of cases that are available to study within a reasonable time‐frame. The sheer extent of the COVID‐19 pandemic obviously overcomes this, and work such as that presented here are first steps in interrogating these links.

The pattern of skin and muscle autoantibodies is intriguing, and further studies are needed to elucidate the antigenic target and the clinical significance of these autoantibodies. One interesting possibility is the desmoglein (DSG) family, and DSG1 and 3 are found in the autoimmune blistering pemphigus disorders. While oral ulceration and blistering has been described in COVID‐19 (16, 17)) it is by no means a commonly reported feature in large clinical studies such as ISARIC4C (Coronavirus Clinical Characterisation Consortium) (18).

There are a number of limitations to this observational, hypothesis‐generating study. First, we have not investigated for or demonstrated a direct pathogenic link between COVID‐19 infection and clinical autoimmunity, and this will be the focus of further research. However, the profile of autoantibodies observed during and after COVID‐19 infection differed from that observed in patients on ITU for other reasons, despite more than 50% of these patients suffering from an infectious pathology, most commonly pneumonia. We cannot exclude the possibility that these observations are a non‐specific consequence of severe respiratory viral infection, and the recruitment of further cohorts of patients (e.g. severe influenza) will address this possibility. Secondly, the COVID‐19 cohorts were recruited prior to dexamethasone and tocilizumab becoming the standard of care for severe COVID‐19. Only a minority of patients in each of our study groups received corticosteroids. Whether these treatments will affect the prevalence or pattern of autoantibodies detectable following COVID‐19 requires further study. Lastly, indirect immunofluorescence only provides a qualitative assessment of the presence or absence of autoantibodies and does not provide quantitative assessment or confirmation of the exact antigenic target, which will need further study. The advantage of the testing strategy in this study is that these are all clinically relevant and standardized assays, so the staining patterns are well described.

This study has explored the relationship between COVID‐19 and autoantibody generation. Future studies are required to confirm whether this is a SARS‐CoV‐2 specific effect or due to non‐specific inflammatory effects of severe respiratory illness. The clinical relevance of these autoantibodies needs to be determined; the profile of autoantibodies observed may help to direct the specific history, examination and investigations necessary in COVID‐19 follow‐up clinics. Together, these would facilitate our understanding of whether or not autoantibodies contribute to the myriad of post‐COVID presentations described.

## CONFLICT OF INTERESTS

Mark T. Drayson reports personal fees from Abingdon Health, outside the submitted work. All other authors declare no competing interests.

## Ethical Approval

For the acute cohort of ITU patients, surplus anonymized samples from routine clinical testing were used, and for the convalescent ITU patients they were consented in clinic; ethical approval for these groups was granted by the North West‐Preston Research Committee (ref. 20/NW/0240 IRAS Project ID: 282164). The health‐care worker cohort was a random subgroup of COVID antibody‐positive patients from the COvid‐19 COnvalescent immunity study (COCO) study approved by the London–Camden and Kings Cross Research Ethics Committee (ref. 20/HRA/1817).

## Supporting information

Table S1‐S2

## Data Availability

The data that supports the findings of this study are available in the supplementary material of this article.
