# Supplementary material for: Establishing the prevalence of common tissue‐specific autoantibodies following severe acute respiratory syndrome coronavirus 2 infection
Source: Clin Exp Immunol. 2021 Jun 13;205(2):99–105. doi: 10.1111/cei.13623 (PMC8239842; doi:10.1111/cei.13623)
Supplement: Supplementary file 1 — Table S1‐S2 [file CEI-205-99-s001.docx]

**Supplementary Table 1: Study participants**

| **Participant** | **Study**  **Group** | **Sex** | **Age** | **Time from symptom onset** | **Disease** | **Notes** |
| --- | --- | --- | --- | --- | --- | --- |
| 1 | 1 | Male | 38 | n/a | Pneumonia, Alcohol and drug excess | |
| 2 | 1 | Female | 61 | n/a | Pneumonia, pulmonary oedema, Granulomatosis with polyangitis | |
| 3 | 1 | Male | 68 | n/a | Epidural abscess | |
| 4 | 1 | Male | 76 | n/a | Aspiration pneumonia on background of multifocal motor neuropathy | |
| 5 | 1 | Male | 49 | n/a | Enterococcal sepsis, subdural haematoma | |
| 6 | 1 | Male | 81 | n/a | Multiorgan failure | |
| 7 | 1 | Male | 29 | n/a | Renal infarction | |
| 8 | 1 | Male | 64 | n/a | Major trauma | |
| 9 | 1 | Female | 87 | n/a | Pneumonia, acute respiratory distress syndrome | |
| 10 | 1 | Male | 64 | n/a | Pneumonia, acute kidney injury | |
| 11 | 1 | Female | 76 | n/a | Rhabdomyolysis, ischaemic stroke | |
| 12 | 1 | Female | 50 | n/a | Variceal bleed, alcohol misuse | |
| 13 | 1 | Female | 33 | n/a | Acute asthma | |
| 14 | 1 | Male | 49 | n/a | Aortic root abscess, aortic valve replacement | |
| 15 | 1 | Female | 74 | n/a | Aortic valve replacement, disseminated intravascular coagulation | |
| 16 | 1 | Male | 50 | n/a | Influenza, Staphylococcal pneumonia | |
| 17 | 1 | Female | 58 | n/a | CMV pneumonitis, pneumonia, HHV8 encephalitis | |
| 18 | 1 | Female | 51 | n/a | Infective arthritis, acute kidney injury | |
| 19 | 1 | Female | 88 | n/a | Pneumonia, chronic inflammatory demyelinating polyneuropathy | |
| 20 | 1 | Male | 70 | n/a | Acute renal failure | |
| 21 | 1 | Female | 34 | n/a | HELLP syndrome, E coli sepsis, liver abscess | |
| 22 | 1 | Female | 30 | n/a | Anaphylaxis | |
| 23 | 1 | Male | 56 | n/a | Pneumonia | |
| 24 | 1 | Male | 67 | n/a | Out of hospital cardiac arrest | |
| 25 | 1 | Female | 76 | n/a | Peritonitis, large bowel perforation | |
| 26 | 1 | Female | 68 | n/a | Neuroleptic malignant syndrome | |
| 27 | 1 | Female | 37 | n/a | Major trauma | |
| 28 | 1 | Male | 74 | n/a | Pneumonia | |
| 29 | 1 | Male | 75 | n/a | ARDS, ventilator associated pneumonia post oesophagectomy | |
| 30 | 1 | Male | 67 | n/a | Type 2 respiratory failure | |
| 31 | 1 | Male | 59 | n/a | Upper gastrointestinal bleed, multiorgan failure | |
| 32 | 1 | Female | 26 | n/a | Acute kidney injury, placental abruption, haemorrhagic shock | |
| 33 | 2 | Male | 64 | 34 | COVID-19 |  |
| 34 | 2 | Male | 60 | 20 | COVID-19 |  |
| 35 | 2 | Male | 60 | 23 | COVID-19 |  |
| 36 | 2 | Male | 47 | 20 | COVID-19 |  |
| 37 | 2 | Male | 50 | 25 | COVID-19 |  |
| 38 | 2 | Male | 42 | 17 | COVID-19 |  |
| 39 | 2 | Male | 50 | 24 | COVID-19 |  |
| 40 | 2 | Male | 67 | 13 | COVID-19 | Ankylosing spondylitis and colitis, not on treatment |
| 41 | 2 | Male | 60 | 18 | COVID-19 |  |
| 42 | 2 | Male | 52 | 16 | COVID-19 | Previous reactive arthritis (2003) with 1/40 ANA |
| 43 | 2 | Male | 71 | 14 | COVID-19 |  |
| 44 | 2 | Male | 39 | 25 | COVID-19 |  |
| 45 | 2 | Male | 21 | 14 | COVID-19 | Primary immunodeficiency |
| 46 | 2 | Female | 51 | 20 | COVID-19 |  |
| 47 | 2 | Male | 71 | 16 | COVID-19 |  |
| 48 | 2 | Female | 44 | 21 | COVID-19 |  |
| 49 | 2 | Male | 69 | 12 | COVID-19 |  |
| 50 | 2 | Female | 56 | 13 | COVID-19 |  |
| 51 | 2 | Male | 55 | 22 | COVID-19 |  |
| 52 | 2 | Male | 51 | 26 | COVID-19 |  |
| 53 | 2 | Male | 58 | 21 | COVID-19 |  |
| 54 | 2 | Male | 49 | 10 | COVID-19 |  |
| 55 | 2 | Male | 52 | 30 | COVID-19 |  |
| 56 | 2 | Male | 61 | 17 | COVID-19 |  |
| 57 | 2 | Female | 63 | 13 | COVID-19 |  |
| 58 | 3 | Female | 55 | 112 | COVID-19 |  |
| 59 | 3 | Male | 48 | 105 | COVID-19 |  |
| 60 | 3 | Male | 47 | 118 | COVID-19 |  |
| 61 | 3 | Male | 56 | 98 | COVID-19 |  |
| 62 | 3 | Male | 45 | 100 | COVID-19 |  |
| 63 | 3 | Male | 51 | 117 | COVID-19 |  |
| 64 | 3 | Male | 37 | 82 | COVID-19 |  |
| 65 | 3 | Male | 51 | 118 | COVID-19 |  |
| 66 | 3 | Female | 51 | 116 | COVID-19 |  |
| 67 | 3 | Male | 62 | 118 | COVID-19 |  |
| 68 | 3 | Male | 56 | 115 | COVID-19 |  |
| 69 | 3 | Female | 67 | 130 | COVID-19 |  |
| 70 | 3 | Male | 62 | 115 | COVID-19 |  |
| 71 | 3 | Male | 56 | 147 | COVID-19 |  |
| 72 | 3 | Male | 49 | 147 | COVID-19 |  |
| 73 | 3 | Male | 54 | 178 | COVID-19 | Rheumatoid arthritis (CCP+ RF+, ANA-) |
| 74 | 3 | Female | 57 | 169 | COVID-19 |  |
| 75 | 3 | Female | 70 | 204 | COVID-19 |  |
| 76 | 3 | Male | 43 | 153 | COVID-19 |  |
| 77 | 3 | Male | 45 | 176 | COVID-19 |  |
| 78 | 3 | Male | 60 | 125 | COVID-19 | Type 1 diabetes, seronegative inflammatory arthritis |
| 79 | 3 | Male | 37 | 173 | COVID-19 |  |
| 80 | 3 | Male | 65 | 179 | COVID-19 | Renal transplant (primary renal disease unknown) |
| 81 | 3 | Male | 33 | 165 | COVID-19 |  |
| 82 | 3 | Male | 49 | 172 | COVID-19 |  |
| 83 | 3 | Male | 65 | 180 | COVID-19 |  |
| 84 | 3 | Male | 69 | 172 | COVID-19 |  |
| 85 | 3 | Female | 54 | 151 | COVID-19 |  |
| 86 | 3 | Male | 50 | 161 | COVID-19 |  |
| 87 | 3 | Male | 62 | 192 | COVID-19 |  |
| 88 | 3 | Male | 22 | 170 | COVID-19 | Primary immunodeficiency |
| 89 | 3 | Male | 63 | 166 | COVID-19 |  |
| 90 | 3 | Male | 46 | 148 | COVID-19 |  |
| 91 | 3 | Male | 61 | 176 | COVID-19 |  |
| 92 | 3 | Male | 56 | 163 | COVID-19 |  |
| 93 | 4 | Female | 39 | 45 | COVID-19 |  |
| 94 | 4 | Female | 51 | 41 | COVID-19 |  |
| 95 | 4 | Female | 35 | 38 | COVID-19 |  |
| 96 | 4 | Female | 43 | 47 | COVID-19 |  |
| 97 | 4 | Female | 63 | 43 | COVID-19 |  |
| 98 | 4 | Female | 41 | 34 | COVID-19 |  |
| 99 | 4 | Female | 50 | 29 | COVID-19 |  |
| 100 | 4 | Male | 45 | 47 | COVID-19 |  |
| 101 | 4 | Female | 27 | 32 | COVID-19 |  |
| 102 | 4 | Female | 49 | 46 | COVID-19 |  |
| 103 | 4 | Female | 32 | 42 | COVID-19 |  |
| 104 | 4 | Female | 57 | 37 | COVID-19 |  |
| 105 | 4 | Female | 51 | 26 | COVID-19 |  |
| 106 | 4 | Unknown | 36 | 30 | COVID-19 |  |
| 107 | 4 | Male | 45 | 37 | COVID-19 |  |
| 108 | 4 | Female | 31 | 22 | COVID-19 |  |
| 109 | 4 | Female | 49 | 32 | COVID-19 |  |
| 110 | 4 | Male | 49 | 47 | COVID-19 |  |
| 111 | 4 | Female | 51 | 45 | COVID-19 |  |
| 112 | 4 | Female | 57 | 30 | COVID-19 |  |
| 113 | 4 | Female | 29 | 39 | COVID-19 |  |
| 114 | 4 | Male | 34 | 39 | COVID-19 |  |
| 115 | 4 | Female | 48 | 38 | COVID-19 |  |
| 116 | 4 | Female | 41 | 32 | COVID-19 |  |

**Supplementary Table 2: Summary of initial serum dilutions for indirect immunofluorescence assays and typical clinical association of autoantibodies**

| **Tissue** | **Isotype** | **Method** | **Starting dilution** | **Threshold for strong positive** | **Clinical association** |
| --- | --- | --- | --- | --- | --- |
| Adrenal | IgG | Indirect immunofluorescence using Inova kit | 1/5 | Qualitative | Addison’s Disease |
| Autoimmune encephalitis screen | IgG | Indirect immunofluorescence using Euroimmun kit | 1/10 | Qualitative | Autoimmune encephalitis,  paraneoplastic encephalitis |
| ANA | IgG | Indirect immunofluorescence using Inova kit | 1/100 | Equal to or greater than 1/100 | Non-specific; high sensitivity for systemic lupus erythematosus, drug induced lupus |
| ANCA | IgG | Indirect immunofluorescence using Inova kit | 1/20 | Equal to or greater than 1/40 | Small vessel vasculitis |
| Cardiac | IgG | Indirect immunofluorescence using Inova kit | 1/5 | Qualitative | Dressler’s syndrome |
| Endomysial | IgA | Indirect immunofluorescence using Inova kit | 1/10 | Qualitative | Coeliac disease |
| Epidermal (IC) | IgG | Indirect immunofluorescence using Inova kit | 1/10 | Qualitative | Pemphigus vulgaris |
| Epidermal  (BM) | IgG | Indirect immunofluorescence using Inova kit | 1/10 | Qualitative | Bullous pemphigoid |
| Islet cell antibodies | IgG | Indirect immunofluorescence using Inova kit | 1/5 | Qualitative | Type 1 diabetes |
| Purkinje cell antibodies | IgG | Indirect immunofluorescence using Inova kit | 1/50 | Qualitative | Paraneoplastic encephalitis |
| Smooth muscle antibodies | IgG | Indirect immunofluorescence using Inova kit | 1/20 | Equal to or greater than  1/40 | Autoimmune hepatitis (type 1) |
| Gastric parietal cell antibodies | IgG | Indirect immunofluorescence using Inova kit | 1/20 | Qualitative | Atrophic gastritis  Pernicious anaemia |
| Skeletal muscle antibodies | IgG | Indirect immunofluorescence using Inova kit | 1/5 | Qualitative | Myasthenia gravis (typically when associated with thymoma) |
